# Genome-Wide Association Study for Serum Complement C3 and C4 Levels in Healthy Chinese Subjects

**DOI:** 10.1371/journal.pgen.1002916

**Published:** 2012-09-13

**Authors:** Xiaobo Yang, Jielin Sun, Yong Gao, Aihua Tan, Haiying Zhang, Yanling Hu, Junjie Feng, Xue Qin, Sha Tao, Zhuo Chen, Seong-Tae Kim, Tao Peng, Ming Liao, Xiaoling Lin, Zengfeng Zhang, Minzhong Tang, Li Li, Linjian Mo, Zhengjia Liang, Deyi Shi, Zhang Huang, Xianghua Huang, Ming Liu, Qian Liu, Shijun Zhang, Jeffrey M. Trent, S. Lilly Zheng, Jianfeng Xu, Zengnan Mo

**Affiliations:** 1 Department of Occupational Health and Environmental Health, School of Public Health, Guangxi Medical University, Nanning, Guangxi, China; 2 Center for Genomic and Personalized Medicine, Guangxi Medical University, Nanning, Guangxi, China; 3 Center for Cancer Genomics, Wake Forest University School of Medicine, Winston-Salem, North Carolina, United States of America; 4 Fudan-VARI Center for Genetic Epidemiology, School of Life Sciences, Fudan University, Shanghai, China; 5 Medical Scientific Research Center, Guangxi Medical University, Nanning, Guangxi, China; 6 Department of Clinical Laboratory, The First Affiliated Hospital of Guangxi Medical University, Nanning, Guangxi, China; 7 Department of Hepatobiliary Surgery, The First Affiliated Hospital of Guangxi Medical University, Nanning, Guangxi, China; 8 Laboratory of Genomic Diversity, National Cancer Institute, National Institutes of Health, Frederick, Maryland, United States of America; 9 Department of Microbiology, School of Preclinical Medicine, Guangxi Medical University, Nanning, Guangxi, China; 10 College of Life Science and Bioengineering, Beijing University of Technology, Beijing, China; 11 Institute of Urology and Nephrology, The First Affiliated Hospital of Guangxi Medical University, Nanning, Guangxi, China; 12 Medical Examination Center, Fangchenggang First People's Hospital, Fangchenggang, Guangxi, China; 13 Medical Examination Center, Guigang People's Hospital, Guigang, Guangxi, China; 14 Medical Examination Center, Yulin First People's Hospital, Yulin, Guangxi, China; 15 Institute of Cardiovascular Diseases, The First Affiliated Hospital of Guangxi Medical University, Nanning, Guangxi, China; 16 Center for Genetic Epidemiology, Van Andel Research Institute, Grand Rapids, Michigan, United States of America; 17 Fudan University Institute of Urology, Huashan Hospital, Fudan University, Shanghai, China; National Institute of Genetics, Japan

## Abstract

Complement C3 and C4 play key roles in the main physiological activities of complement system, and their deficiencies or over-expression are associated with many clinical infectious or immunity diseases. A two-stage genome-wide association study (GWAS) was performed for serum levels of C3 and C4. The first stage was conducted in 1,999 healthy Chinese men, and the second stage was performed in an additional 1,496 subjects. We identified two SNPs, rs3753394 in *CFH* gene and rs3745567 in *C3* gene, that are significantly associated with serum C3 levels at a genome-wide significance level (*P = *7.33×10^−11^ and *P = *1.83×10^−9^, respectively). For C4, one large genomic region on chromosome 6p21.3 is significantly associated with serum C4 levels. Two SNPs (rs1052693 and rs11575839) were located in the MHC class I area that include *HLA-A*, *HLA-C*, and *HLA-B* genes. Two SNPs (rs2075799 and rs2857009) were located 5′ and 3′ of *C4* gene. The other four SNPs, rs2071278, rs3763317, rs9276606, and rs241428, were located in the MHC class II region that includes *HLA-DRA*, *HLA-DRB*, and *HLA-DQB* genes. The combined *P*-values for those eight SNPs ranged from 3.19×10^−22^ to 5.62×10^−97^. HBsAg-positive subjects have significantly lower C3 and C4 protein concentrations compared with HBsAg-negative subjects (*P*<0.05). Our study is the first GWAS report which shows genetic components influence the levels of complement C3 and C4. Our significant findings provide novel insights of their related autoimmune, infectious diseases, and molecular mechanisms.

## Author Summary

The complement system plays important roles in the innate and adaptive immune functions. C3 and C4 participate in almost all physiological activities and activated pathways as key complement members and host defense proteins. Identifying the genes that influence serum levels of C3 and C4 may help to elucidate the factors and mechanisms underlying the complement system. The genome-wide association studies (GWAS) have shown great success in revealing robust associations in both quantitative and qualitative traits. In this study, we performed a two-stage GWAS in a large cohort from the Chinese male population to examine the roles of common genetic variants on serum C3 and C4 levels. Our research identified genetic determinants associated with the quantitative levels of C3 and C4. Overall, our study highlights an intricate regulation of complement levels and potentially reveals novel mechanisms that may be followed up with additional functional studies.

## Introduction

The complement has been recognized as one pivotal part of innate and adaptive immune system, and it had three well-known physiologic activities, including host defense against infection, bridging interface between innate and adaptive immunity, and disposal of waste immune complexes or apoptotic cells [1], [2]. There are approximately 30 serum complement proteins, which can be activated by classical, alternative, and lectin pathways [3]. Among these complement members, C3 and C4 are almost involved in all physiological activities and activated pathways, and exert their powerful roles as host defense proteins [4]. Their abnormal expression, especially, complement deficiency and dysfunction, is associated with the pathogenesis of numerous inflammatory and autoimmune disorder diseases, such as systemic lupus erythematosus (SLE), rheumatoid arthritis, asthma [1], [5]. C4 deficiency also has been reported to be associated with SLE, immune complex glomerulonephritis, virus infection, regardless of ethnicity background [6], [7], [8].

Both Serum C3 and C4 levels are heritable traits even in individuals without immune diseases such as SLE. Recent reports also showed that the heritability for serum C3 and C4 were 39.6% and 45.4%, respectively, based on Bayesian variance components model [9], [10]. Therefore, it is important to identify the genetic loci which influence the serum levels of C3 and C4. In this study, we carried out a two-stage Genome-Wide Association Study (GWAS) to identify novel loci that are associated with the serum levels of C3 and C4 in a healthy Chinese male population of 3,495 subjects.

## Methods

### Study samples

A total of 2,012 men from the Fangchenggang Area Male Health and Examination Survey (FAMHES) participated in the stage 1 of GWAS study as healthy volunteers. The FAMHES project was conducted in Fangchenggang city, Guangxi, southern China in 2009. A total of 4,303 Chinese men ranged from age 17 to 88 years-old were recruited [11]. The subjects used in our stage 1 were limited to age 20 to 69 years-old, and were all self-reported southern Chinese Han ethnicity. The replication subjects in the stage 2 included 1,496 men age 20 to 70 years-old, who were recruited in conjunction with health examinations that were performed at three collaborating hospitals in Guangxi, China.

Written informed consents to participate in the present study were obtained from all of the subjects. Standardized health questionnaires were then used through a face-to-face interview conducted by trained physicians. Collected data included demographic, lifestyle characteristics (smoking, alcohol consumption), health status, family history, and medical histories. The subjects who self-reported with diabetes mellitus, coronary heart disease, stroke, hyperthyroidism, rheumatoid arthritis, and tumors were excluded from the study population. The detected samples with abnormal liver function (ALT) were also excluded. The study was approved by the Ethics and Human Subject Committee of Guangxi Medical University.

### Measurement of complement C3 and C4

Overnight fasting venous blood specimens were drawn between 7:00 and 10:00 am, and serum samples were extracted and stored at −80°C, until required for detection in the laboratory. Both serum C3 and C4 were measured with Immunoturbidimetric methods on the HITACHI 7600 biochemistry analyzer (Hitachi Corp, Tokyo, Japan), using complement C3 and C4 assay kits (Zhicheng Bio Company, Shanghai, China), and their inter-assay coefficients of variation were 2.1% and 2.5%, respectively.

### SNP genotyping

Two different platforms were used for SNP genotyping. The Illumina Omini one platform was used for the genome-wide assay of samples in stage 1. For SNPs that were followed in the 2nd stage, the iPLEX of Sequenom platform was used for genotyping (Sequenom, Inc., San Diego, CA). Polymerase chain reaction (PCR) and extension primers were designed using MassARRAY Assay Design 3.1 software (Sequenom, Inc., San Diego, CA). Genotyping procedures were performed according to the manufacturer's iPLEX Application Guide (Sequenom Inc. SanDiego, CA). All genotyping reactions were performed in 384-well plates. Each plate included a duplicate for three or four subjects selected at random, as well as six to nine negative controls in which water was substituted for DNA. The average concordance rate was 99.8%.

### CNV analysis for *C4* gene

To evaluate the *C4* copy number variations (CNV), quantitative PCR (qPCR) was performed using SYBR green chemistry on the 100 randomly selected subjects from Stage 1 population. Three pairs of specific primers were designed at the front, middle rear position of *C4* gene. Two pairs of internal primers based on *RPP14* and *ALB* genes were selected to normalize the nonspecific error. Relative gene dosage determination was carried out using an ABI 7900 Real time PCR System. Total 12 µl PCR reaction mixture contained 3 µl free water, 6 µl 2×SYBR (ABI, Part no. 4304886), 1.8 µl primers, and 1.2 µl genomic DNA template. The delta-delta Ct method was used to calculate the copy numbers of *C4* gene. The average *C4* copy number was set at 4 based on previous reports.

### Statistical analysis

Quality Control (QC) procedures were first applied to 2,012 individuals that were genotyped using the Illumina Omni one platform. A total of 1,999 individuals passed the call rate of 95% and were used in the final statistical analysis. We then applied the following QC criteria to filter SNPs: *P*<0.001 for the Hardy-Weinberg Equilibrium (HWE) test, minor allele frequency (MAF) <0.01, and genotype call rate <95%. A total of 709,211 SNPs remained in the final analysis. The IMPUTE computer program was then used to infer the genotypes of SNPs (e.g. SNPs catalogued in Hapmap Phase II CHB population release #24) in the genome that were not directly genotyped [12]. A posterior probability of >0.90 was applied to call genotypes that were imputed from IMPUTE software. A total of 1,940,245 SNPs remained in our final analysis after applying the same QC criteria, as mentioned above.

Serum C4 levels were logarithmic transformed to normalize the distribution. The associations of the SNPs with serum C3 and C4 levels were evaluated using a linear regression model assuming additive effects of the alleles (0, 1 and 2). In the regression models, the age, logBMI were adjusted as covariates for both C3 and C4, and smoking also adjusted for C3.The PLINK software package was used to perform this statistical analysis [13]. The EIGENSTRAT software was applied for the evaluation of population stratification by a principal component approach [14]. The top two eigenvectors were adjusted as covariates in the linear regression analysis. Quantile-Quantile plots were generated using R package. For regions with multiple SNPs that were significant at a genome-wide significant level of 5×10^−8^, a stepwise (forwards - backwards) regression modeling was performed to select the independent SNPs to be confirmed in the 2^nd^ stage. Firstly, the most significant SNP within each of the associated region with C3 (1q21.3 and 19p13) and C4 (MHC region in 6p21.3) was entered into the linear regression model. Secondly, the step-wise procedure iteratively added SNPs (forwards) that reach genome-wide significant level in the single SNP analysis (with adjustment of covariates). A SNP remains in the model if goodness-of-fit (the Arkaike Information Criterion (AIC) metric) improves. A SNP that is already in the model can also be removed if the AIC improves by doing so. The stepwise procedure was performed by SAS 9.2 (Cary, NC). The combined analysis of two-stage data was performed using a linear regression, adjusting for the covariates and stage information. ANOVA analysis was used to evaluate the difference of serum C4 levels among the varied *C4* gene dose groups.

## Results

### General subjects characters

The first stage was composed of 1999 healthy Chinese men, and the second stage with 1496 male subjects. No significant difference was found between samples of the first and the second stage (Table 1) regarding to the mean age, smoking distribution and mean body mass index, *P*>0.05, respectively. The frequency distribution of C3 and C4 levels among the stage 1 healthy population were shown in the Figure S1. An analysis of the correlation between serum levels of C3 and C4 revealed a significantly positive relationship (r = 0.521, *P*<0.01).

**Table 1:** General characteristic of the two-stage GWAS study subjects.

Characteristic	First stage	Second stage	*P* value^a^^,^ b
N	1999	1496	
Age (years)	37.54±11.10	37.31±10.80	0.54
Smoking			
Yes	1015 (50.8)	771 (51.5)	0.66
No	984 (49.2)	725 (48.5)	
Body mass index (kg.m^−2^)	23.31±3.44	23.46±3.34	0.18

a t-Test was used to compare means between the first and the second stages.

b Chi-square test was used to compare the differences in categorized variables.

In stage 1, limited population stratification was observed (Figure S2). In addition, we did not observe any evidence for the systematic bias of the association for either C3 or C4 phenotype, as indicated by the Quantile-Quantile (Q-Q) plots (Figure S3). The inflation factor was estimated to be 1.046 and 1.054 for C3 and C4, respectively.

### GWAS for serum C3 levels

**Figure 1:**
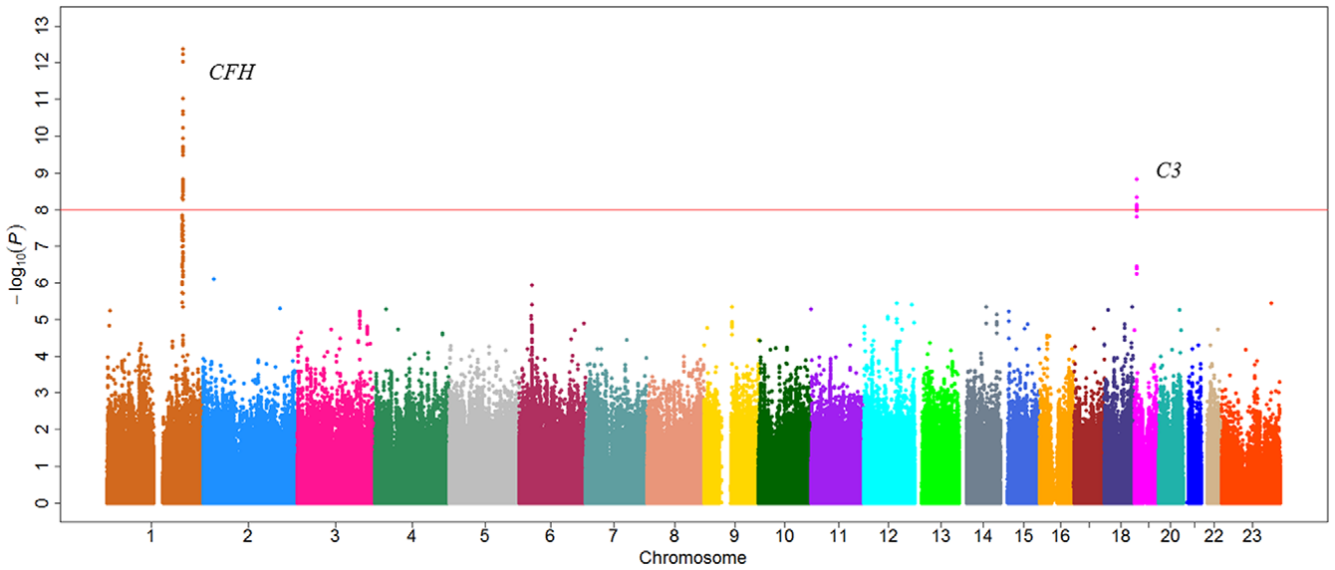
Manhattan plot of genome-wide association analyses for C3 level. X-axis shows chromosomal positions. Y-axis shows –log_10_ *P*-values from linear regression adjusted for age, smoking, and logBMI. The horizontal solid line indicates the preset threshold of *P* = 5×10^−8^.

For C3 level, there are two genomic loci that were significant at a *P-*value cutoff of 5×10^−8^, including one loci on 1q31.3 and one loci on chromosome 19p13.3, respectively (Figure 1 and Figure S4). For the region on 1q31.3, a total of four SNPs reached a *P-*value cutoff of 10^−8^. Rs3753394 was the only SNP that remained in the stepwise model selection procedure. Therefore, only rs3753394 was selected to be followed up in stage 2. For the region on chr19p13.3, the only SNP (rs3745567) that reached a *P-*value of 5×10^−8^ was followed in stage 2. In stage 2, both rs3753394 and rs3745567 were confirmed to be significantly associated with C3 levels at a *P* cut-off of 0.025 (Bonferroni correction of 2 tests) (*P* = 1.08×10^−3^ and *P* = 3.58×10^−4^, respectively). The combined analysis of stage 1 and stage 2 data revealed highly significant association with C3 level for rs3753394 and rs374567 (*P* = 7.33×10^−11^ and *P* = 1.83×10^−9^, respectively) (Table 2). The estimated decrease for C3 level for rs3753394 was 0.05 g/l for each additional T allele (SE = 0.01). For rs3745567, the estimated increase for C3 level was 0.08 g/l for each additional T allele (SE = 0.02). In addition, the proportion of variability in the C3 level explained by the two SNPs was 59.0% (R^2^ = 0.59) in our multivariate linear regression model.

**Table 2:** SNPs associated with C3 and C4 levels from two-stage GWAS study in Chinese population.

SNPs	Chr (Position)^a^	Genes	Gene regions	Minor Allele/Major Allele	First Stage (N = 1999)					Second Stage (N = 1496)					Combined (N = 3495)	
					MAF	Mean levels (g/L)			*P* value^c^	MAF	Mean levels (g/L)			*P* value^c^	β(SE)^c^	*P* value^c^
						aa^b^	Aa	AA			aa	Aa	AA			
C3																
rs3753394	1(194887540)	*CFH*	promoter	C/T	0.43	1.16	1.13	1.08	5.79×10^−13^	0.44	1.31	1.26	1.20	1.08×10^−3^	−0.05(0.01)	7.33×10^−11^
rs3745567	19 (6641771)	*C3*	intron	T/C	0.06	0.93	1.06	1.13	9.32×10^−9^	0.07	1.07	1.16	1.26	3.58×10^−4^	0.09(0.01)	1.83×10^−9^
C4^d^																
rs1052693	6 (30984131)	*HLA-A*	3′	G/A	0.36	0.28	0.31	0.34	3.26×10^−10^	0.34	0.29	0.31	0.35	7.48×10^−22^	0.10(0.01)	3.01×10^−48^
rs11575839	6 (31665770)	*HLA-C*	5′	A/G	0.05	0.47	0.40	0.31	9.70×10^−35^	0.06	0.41	0.41	0.32	2.55×10^−20^	−0.23(0.01)	1.22×10^−54^
rs2075799	6 (31886508)	*C4*	5′	T/C	0.20	0.40	0.36	0.30	1.08×10^−49^	0.20	0.47	0.36	0.30	2.53×10^−48^	−0.17(0.01)	5.62×10^−97^
rs2857009	6 (32127724)	*C4*	3′	G/C	0.20	0.28	0.31	0.33	3.46×10^−24^	0.21	0.28	0.31	0.34	3.04×10^−10^	0.08(0.01)	1.43×10^−22^
rs2071278	6 (32273422)	*HLA-DRA*	5′	G/A	0.27	0.38	0.34	0.30	3.48×10^−29^	0.28	0.41	0.35	0.30	7.86×10^−39^	−0.13(0.01)	4.25×10^−72^
rs3763317	6 (32484766)	*HLA-DRB*	5′	C/T	0.37	0.28	0.31	0.35	1.88×10^−17^	0.38	0.28	0.32	0.38	2.10×10^−32^	0.12(0.01)	9.21×10^−66^
rs9276606	6 (32844673)	*HLA-DQB*	3′	T/A	0.15	0.39	0.34	0.31	2.33×10^−14^	0.15	0.39	0.35	0.32	2.90×10^−10^	−0.09(0.01)	3.19×10^−22^
rs241428	6 (32912048)	*HLA-DQB*	3′	G/T	0.08	0.22	0.26	0.33	3.64×10^−12^	0.09	0.18	0.27	0.34	6.99×10^−39^	0.23(0.01)	9.12×10^−83^

a . Genomic position is based on NCBI build 36.

b . aa indicates serum complement levels for homozygous carriers of minor alleles, Aa indicates for heterozygous carriers, and AA indicates for homozygous carriers of major alleles.

c . *P* values andβ-coefficient are based on multi-linear regression analysis for an addictive effect, and adjusted for age, smoking, and logBMI. The combined *P* s are calculated based on the regression model, adjusting for the covariates and stage information.

d . The presented C4 levels were back-transformed.

### GWAS for serum C4 levels

**Figure 2:**
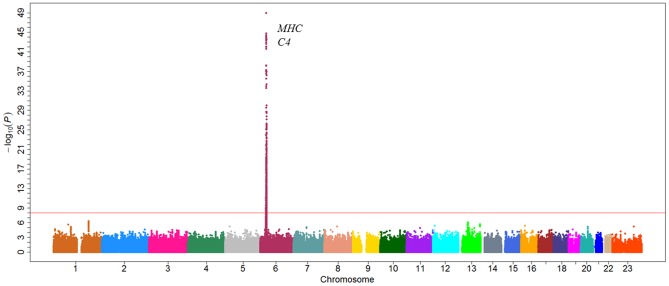
Manhattan plot of genome-wide association analyses for C4 level. X-axis shows chromosomal positions. Y-axis shows −log^10^ *P*values from linear regression adjusted for age and logBMI. C4 level are log-transformed and fit for a normal distribution. The horizontal solid line indicates the preset threshold of *P*=5×10^−8^.

**Figure 3:**
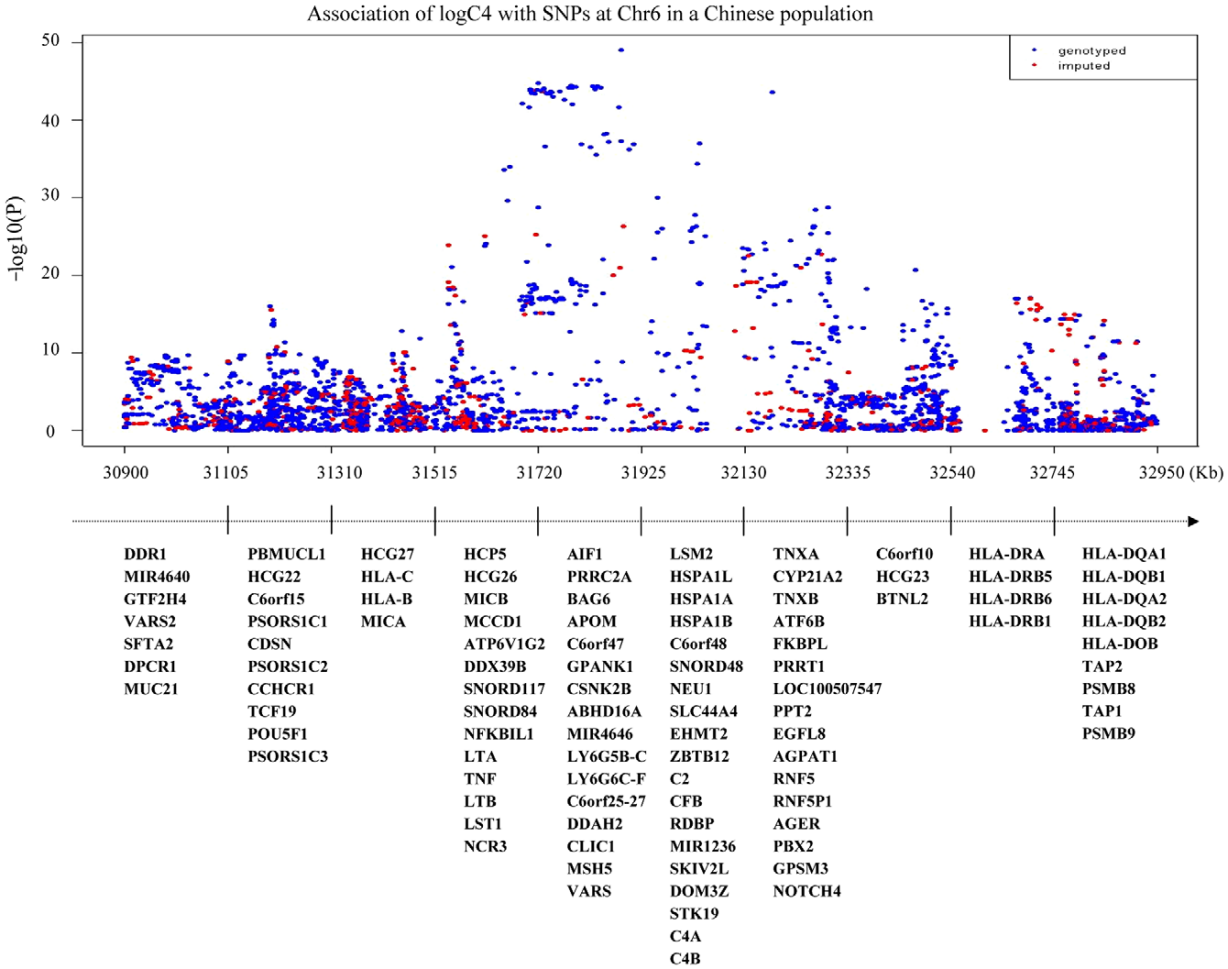
Association of serum C4 levels with SNPs at 6p21.3. X-axis shows base positions from 30,900 Kb to 32,950 Kb. Y-axis shows –log_10_ *P*-values from linear regression adjusted for age and logBMI. C4 level are log-transformed and fit for a normal distribution. The horizontal solid line indicates the preset threshold of *P* = 5×10^−8^. The bottom panels describe all genes in the region.

In the first stage, only one genomic locus was significantly associated with serum level of C4 at a genome-wide significant level (Figure 2). All those SNPs that reached a *P-value* cutoff of 5×10^−8^ are located on the 6p21.3 region. A total of eight independent SNPs were selected based on the stepwise procedure, and were followed in the second stage (Figure S5). These significant SNPs reside in a 2-Mb *MHC* region on chromosome 6p21.3 (Figure 3). Two SNPs (rs1052693 with a *P-*value of 3.26×10^−10^, and rs11575839 with a *P-*value of 9.70×10^−35^) were located in the MHC class I area that include *HLA-A*, *HLA-C*, and *HLA-B* genes. Two SNPs (rs2075799 with a *P-*value of 1.08×10^−49^, and rs2857009 with a *P-*value of 3.46×10^−24^) were located 5′ and 3′ of *C4* gene. The other four SNPs, rs2071278, rs3763317, rs9276606, and rs241428 were located in the MHC class II region that includes *HLA-DRA*, *HLA-DRB*, and *HLA-DQB* genes. In stage 2, all eight SNPS were confirmed to be associated with C4 level at a *P-value* cutoff of 0.006 (Bonferroni correction of eight tests) (*P* value between 3.04×10^−10^ and 2.53×10^−48^). The combined analysis also showed that these SNPs were highly significant associated with C4 levels and reached a genome-wide significant level (*P* value between 3.19×10^−22^ and 5.62×10^−97^) (Table 2). In addition, among the eight SNPs we identified, the range of the increase for C4 level per allele was from 0.08 g/l for rs2857009 (SE = 0.01, *P* = 1.43×10^−22^), and 0.23 g/l for rs1157839 (SE = 0.01, *P* = 1.22×10^−54^). Finally, the proportion of variability in the C4 level explained by the eight SNPs reached 80.5% (R^2^ = 0.805) in our multi-linear regression model.

### Correlation of *C4* gene CNV and serum C4 levels

As shown in Table 3, significant difference of serum C4 level among the varied *C4* gene dose groups were observed, with a *P* value = 3.94×10^−12^. The serum C4 level was significantly higher with each more copy of *C4* gene, with a *P*_trend_ = 5.18×10^−18^. The lowest *C4* gene dose group (N = 3, CNV = 2) only has a mean levels of 0.16±0.03 g/L of C4, which is significantly lower than the group with average number of *C4* gene dose (N = 56, CNV = 4; mean value: 0.33±0.01 g/L, *P* = 5.06×10^−6^). The highest *C4* gene dose group (N = 3, CNV = 7 or 8) had the mean levels of 0.48±0.04 g/L of C4, which is significantly higher than the general dose group, with a *P*-value of 8.24×10^−5^.

**Table 3:** Quantitative variation of serum C4 levels with *C4* gene copy numbers among 100 healthy subjects.

Copy numbers	N	Percent (%)	Serum levels (g/L)	*P* value^a^
2	3	3.0	0.16±0.03	5.06×10^−6^
3	19	19.0	0.26±0.01	1.31×10^−5^
4	56	56.0	0.33±0.01	Refer.
5	16	16.0	0.37±0.01	2.51×10^−2^
6	3	3.0	0.40±0.03	7.29×10^−2^
7,8	3	3.0	0.48±0.04	8.24×10^−5^
Total^b^	100	100.0	0.32±0.08	

a ANOVA analysis was used to compare the serum C4 levels among groups with various copies of *C4* gene dose; Men carrying 4 copies of *C4* gene was used as the reference group.

b Total F value = 17.345, *P* value = 3.94×10^−12^ with degree of freedom of 5.

## Discussion

To our knowledge, our study represents one of the first efforts to identify genetic loci that determine the serum levels of C3 and C4 using a two-stage GWAS approach. We identified three independent genetic loci that were significant at a genome-wide significant level (*P* value<1×10^−8^) in a Chinese population of 3,495 healthy men, with a *P-*value range from 1.83×10^−9^ to 5.62×10^−99^. Those include SNPs rs3745567 (*C3* gene) and rs3753394 (*CFH-CFHR1* gene cluster) and eight independent SNPs in the ∼2 Mb MHC region on chr6 that were significantly associated with serum level of C3 and C4, respectively.

The first confirmed SNP was rs3753394, which is located within the complement factor H (*CFH*) and CFH-related gene (*CFHR1-5*) cluster on 1q31.3. CFH encodes the complement factor H that regulates complement activation on self cells by possessing both cofactor activity for the factor I mediated C3b cleavage, and decay accelerating activity against the alternative pathway C3 convertase [15], [16]. CFHRs contain similar surface recognition region of CFH and there has been evidence that they may play a role in C3b inactivation by binding with C3b [17]. rs3753394 was located in a ∼200 kb LD block region that *CFH*, *CFHR1* and *CFHR3* genes. Thus one possibility is that the genetic variants of rs3753394, may affect the expression or function of one of these three genes, which in turn may cause different modulatory activities towards complement C3 and lead to different levels of C3 proteins. We further used the Genevar (GENE Expression VARiation) database to examine whether rs3753394 is associated with gene expression of CFH and CFH-related genes. rs3753394 was found to be significantly associated with the gene expression level of CFHR1 (*P* = 0.0187) in 156 lymphoblastoid cell line derived from a subset of healthy female twins of the MuTHER resource [18]. No association between rs3753394 and CFH or other CFH family members was found (*P*>0.05). Thus one plausible hypothesis is that the observed rs3753394-C3 association may be causally related with CFHR1 expression.

The second associated SNP with serum level of C3, rs3745567, is located in the intron region of *C3*. Two previous candidate gene studies from Japan and UK SLE families, also showed that genetic variants located in *C3* gene region significantly influence serum C3 levels in SLE patients. The reported SNPs were rs344555 in the 3′ (*P* = 0.007) and rs7951 in the exon 35 (*P* = 0.0024) of *C3* [10], [19]. However, neither rs7951 nor rs344555 was significantly associated with C3 level in our healthy study subjects (*P* = 0.51 and *P* = 0.16, respectively). We then carefully examined the LD structure for CEU, CHB and JPT based on the HapMap Phase II data. The LD structure greatly differed among those three populations in *C3* region (data not shown). Therefore, we may speculate that the previously reported SNP, and the SNP we identified may represent different tagging SNPs for the functional causal SNP in C3 region. The functional variants that reside in *C3* gene region need to be further identified.

For C4, all the eight significant SNPs reside in a 2-Mb *MHC* region on chromosome 6p21. Interestingly, both the complement *C4A* and *C4B* genes whose gene products contribute to serum C4 located in the class III region of MHC on 6p21.3 [8], so the *C4* locus is the most likely candidate with the MHC. Within the MHC region, the *C4* locus (*C4A* and *C4B* paralogues) is the most likely candidate gene that is responsible for serum level of C4. Copy number variations of *C4* gene, as well as gene size polymorphisms of C4 (short or long form) copy number, are known to determine the serum level of C4 [20], [21]. Based on our CNV analysis from the 100 subjects of the stage 1 population, we showed that the total C4 gene dosages were positively correlated with serum levels of C4. Therefore, we can't exclude the possibilities that the SNPs identified in MHC region may be in LDs with *C4* gene copy number or gene size variations. To answer the above question, we evaluated the correlation between C4 gene copy number and the eight SNPs identified. The Pearson correlation coefficients between the eight SNPs and *C4* copy number are moderate, with a range of 0.21 to 0.36 (Table S2). However, seven out of eight SNPs were no longer significant after adjustment for *C4* copy number variation (Table S2). One SNP (rs2857009) still remained significant after adjustment for copy number variation. This suggested that although the majority of SNPs identified may be correlated with copy numbers of C4, one SNP (rs2857009) may confer independent effect on affecting the concentration of C4. The combination of *C4* gene CNV and the SNP information can better explain the genetic components which influence the complement C4 variation. However, we would also like to point out that the above results are based on a relatively small sample size, future studies with a larger sample size is needed to confirm our finding.

Since the identified C4-associated SNPs were located in the MHC region, it will be interesting to know the relationship between those SNPs and known Human Leukocyte Antigens (HLA) alleles. We evaluated whether those eight SNPs and their perfect proxies (r^2^ = 1 based on HapMap 3 plus 1,000 genome project) tag the known HLA alleles, based on the HLA and SNP haplotype map developed by de Bakker et al [22]. However, none of the eight SNPs or their perfect proxies was found to tag the known HLA alleles in CHB population. Two reasons may potentially explain it. First, the number of individuals that were used to construct such map was small (N = 45). Therefore, haplotypes with relatively low frequencies were not detected. In a recent study, a new HLA and SNP haplotype map was available based on a relatively larger population (N = 3,000), which represented an improved haplotype map with much higher resolution [23]. However, this map was currently available for Caucasians only. Second, our study population represents a southern Chinese population. The LD structure of our population may differ from CHB population, which represents a northern Chinese population. The LD structure may even differ significantly in the highly polymorphic MHC region between CHB and our study population. Therefore, a HLA and SNP haplotype map that is specific to this study population may be more informative to infer the LD between the identified C4 associated SNPs and known HLA alleles.

Some confounding factors, such as age, smoking, BMI, and hsCRP, are predictive of the C3 or C4 levels, however, the most significant covariate for C3 is the BMI (*P*  =  2.88×10^−7^) , and age for C4 (*P* = 5.56×10^−11^) in our multivariate linear model which also included novel SNPs identified in this study. Together, those variables could explain more than 90% of variance of C4 level (R^2^>0.90). Serum level of high-sensitivity Chronic Reactive Protein (hsCRP) levels, which is a potential confounder for the acute phase reactants C3 and C4 protein, was also available in our dataset. Further adjustment of hsCRP revealed similar results (Table S1). Considering the high infection rate of hepatitis B virus in the general Chinese population and its potential influence for complement, we furthermore carried out interaction analysis of infection-by-genotype. Although both the C3 and C4 levels in the HBsAg positive group were significantly lower than the negative group in the combined 3495 subjects (1.09±0.21 vs1.18±0.34 for C3 and 0.32±0.09 vs 0.34±0.10 for C4, *P*<0.05, respectively), however, no significant interaction was observed between HBsAg infection and previous identified SNPs in our multivariate linear regression model, *P*>0.05, respectively. The potential cause could be that the synthesis ability of complements was still normal in the liver organ.

In summary, through a two-stage GWAS of 3,495 healthy Chinese male subjects, we identified *CFH* and *C3* loci were significantly associated with serum level of C3, and a ∼2 Mb loci on 6p21.3 region was significantly associated with serum level of C4. The selection of healthy subjects from one centralized resource and the stringent inclusion and exclusion criteria were considered as two of the advantages of our study design. Our study would contribute to the understanding of genetic components that affect individual level of complement variation. It may also shed light on the etiology and molecular mechanisms for C3 and C4 related diseases.

## Supporting Information

Figure S1 Distribution of C3 and C4 levels among the Stage 1 population. The histogram figure showing the frequency of C3 and C4 levels, respectively, and the C4 are logarithmic transformed to normalize the distribution.
(DOC)

Figure S2 Principal component analysis of GWAS sample. HapMap individuals (CHB+JPT, CEU and YRI) for the first two dimensions. The plot presents the top two eigen vectors identified by principal component analysis using Eigenstrat software.
(DOC)

Figure S3 Quantile–quantile plots. Quantile–quantile plot showing the distribution of expected compared to observed –log10 P for the association test results before (red circles) and after genomic control adjustment (blue circles).
(DOC)

Figure S4 Association of serum C3 levels with SNPs at 1q32 and 19p13.3. The top panel presents association results in the first stage of GWAS, the middle panel describes genes in the region based on the UCSC database, and the bottom panel describes LD of SNPs in the region based on the HapMap CHB population (release 24).
(DOC)

Figure S5 Association of serum C3 levels with SNPs at 6p21.3. The top panel presents association results in the first stage of GWAS, the middle panel describes genes in the region based on the UCSC database, and the bottom panel describes LD of SNPs in the region based on the HapMap CHB population (release 24).
(DOC)

Table S1 SNPs associated with C3 and C4 levels from two-stage GWAS study in Chinese population, adjusting for chronic reactive protein (CRP).
(DOC)

Table S2 Correlation estimates between the significant SNPs with C4 copy number variation.
(DOC)
