# Supplementary figures and images for: Genome-Wide Association Study for Serum Complement C3 and C4 Levels in Healthy Chinese Subjects
Source: PLoS Genet. 2012 Sep 13;8(9):e1002916. doi: 10.1371/journal.pgen.1002916 (PMC3441730; doi:10.1371/journal.pgen.1002916)

**Figure S1**

**(A)**


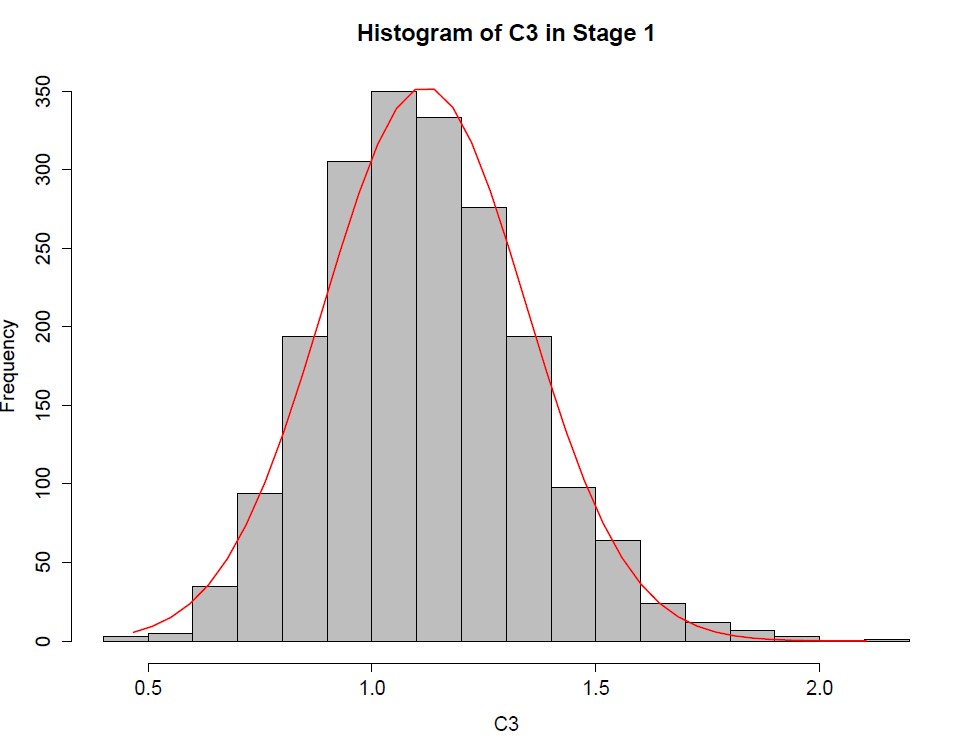


Number of Individuals

**(B)**


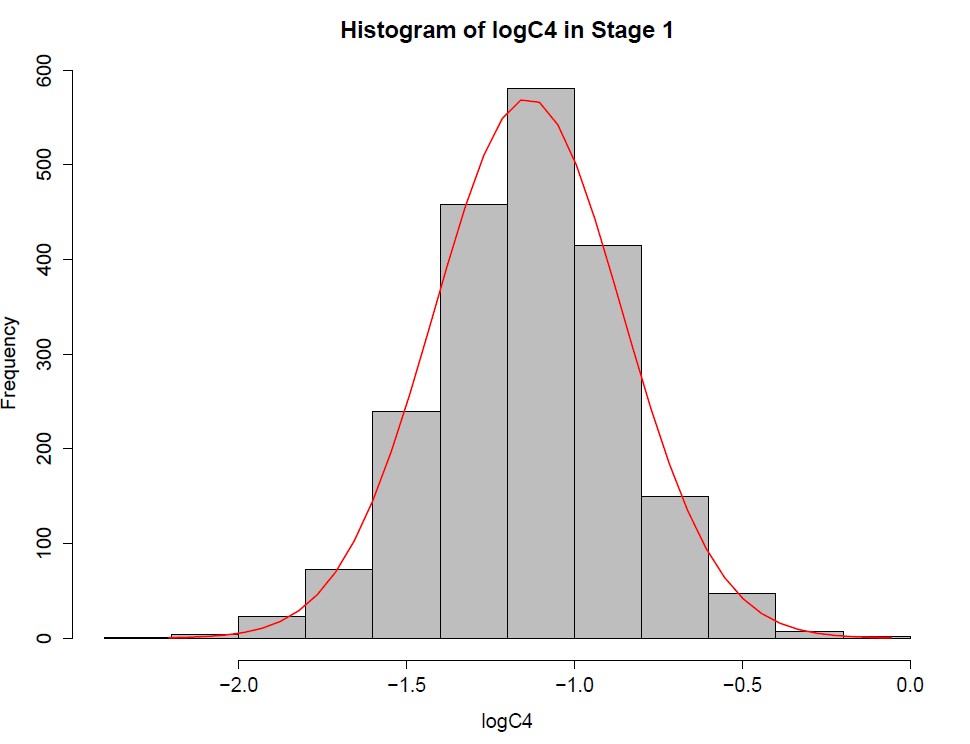


Number of Individuals

Supplement: Figure S1 — Distribution of C3 and C4 levels among the Stage 1 population. The histogram figure showing the frequency of C3 and C4 levels, respectively, and the C4 are logarithmic transformed to normalize the distribution. (DOC) [file pgen.1002916.s001.doc]

**Figure S2.**

**
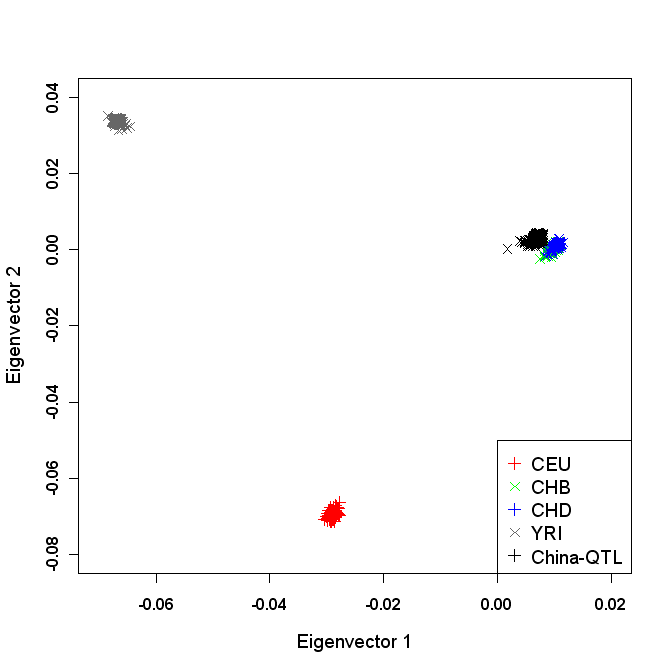
**

Supplement: Figure S2 — Principal component analysis of GWAS sample. HapMap individuals (CHB+JPT, CEU and YRI) for the first two dimensions. The plot presents the top two eigen vectors identified by principal component analysis using Eigenstrat software. (DOC) [file pgen.1002916.s002.doc]

**Figure S3**

**(A)**


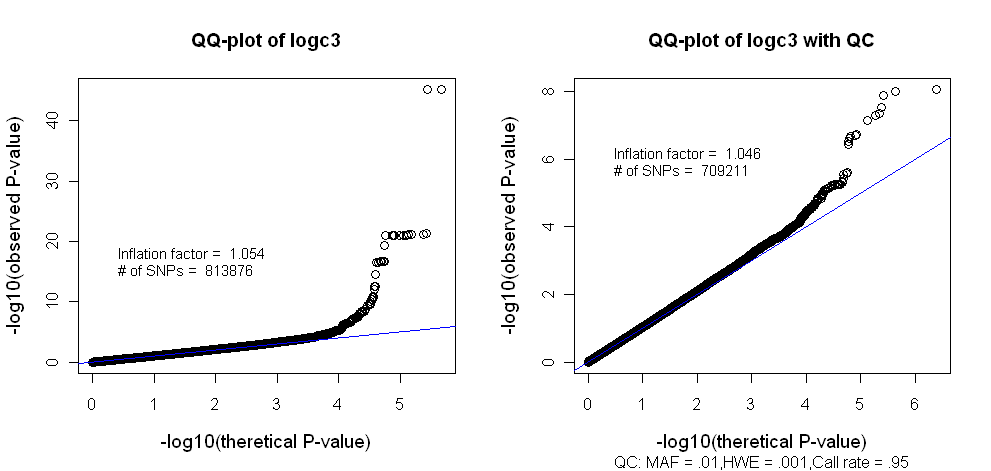


**(B)**


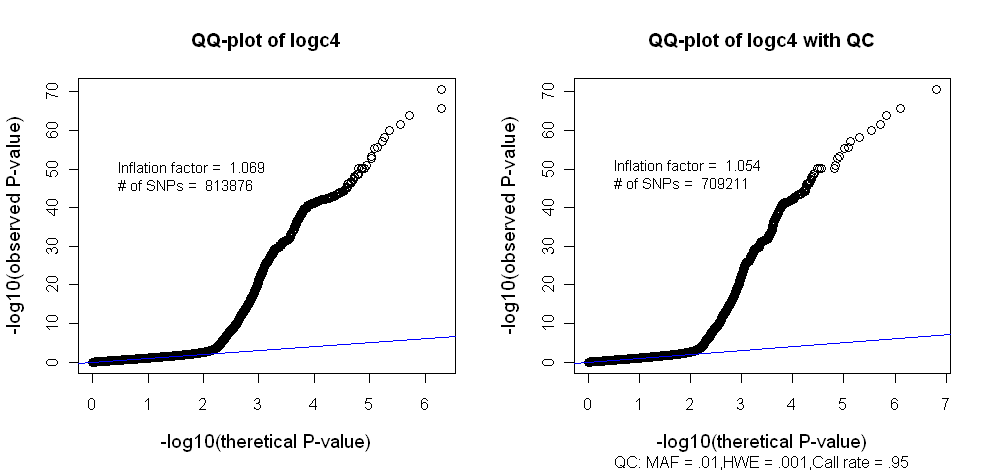

Supplement: Figure S3 — Quantile–quantile plots. Quantile–quantile plot showing the distribution of expected compared to observed –log10 P for the association test results before (red circles) and after genomic control adjustment (blue circles). (DOC) [file pgen.1002916.s003.doc]

**Figure S4**

**(A)**


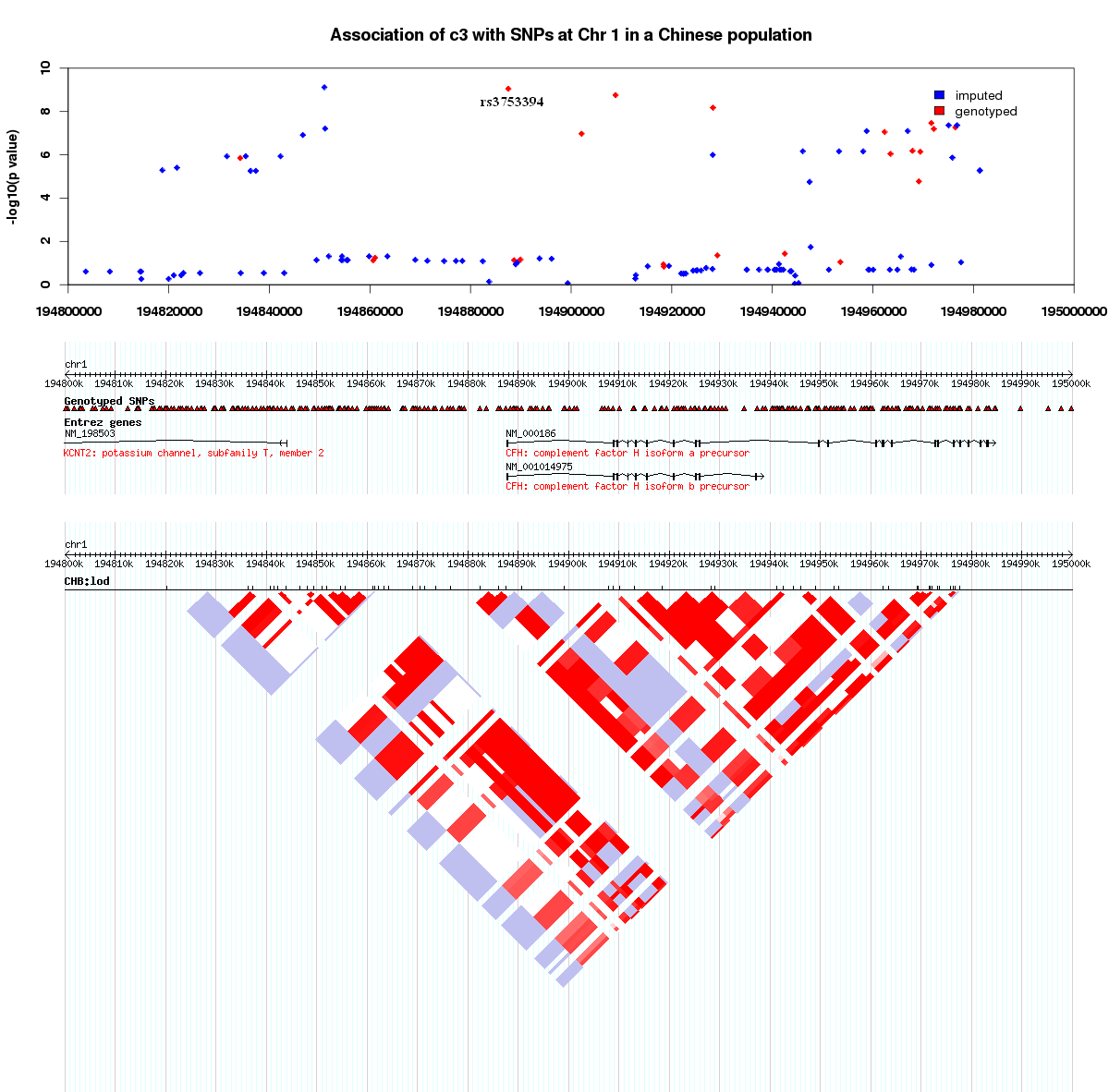


**(B)**


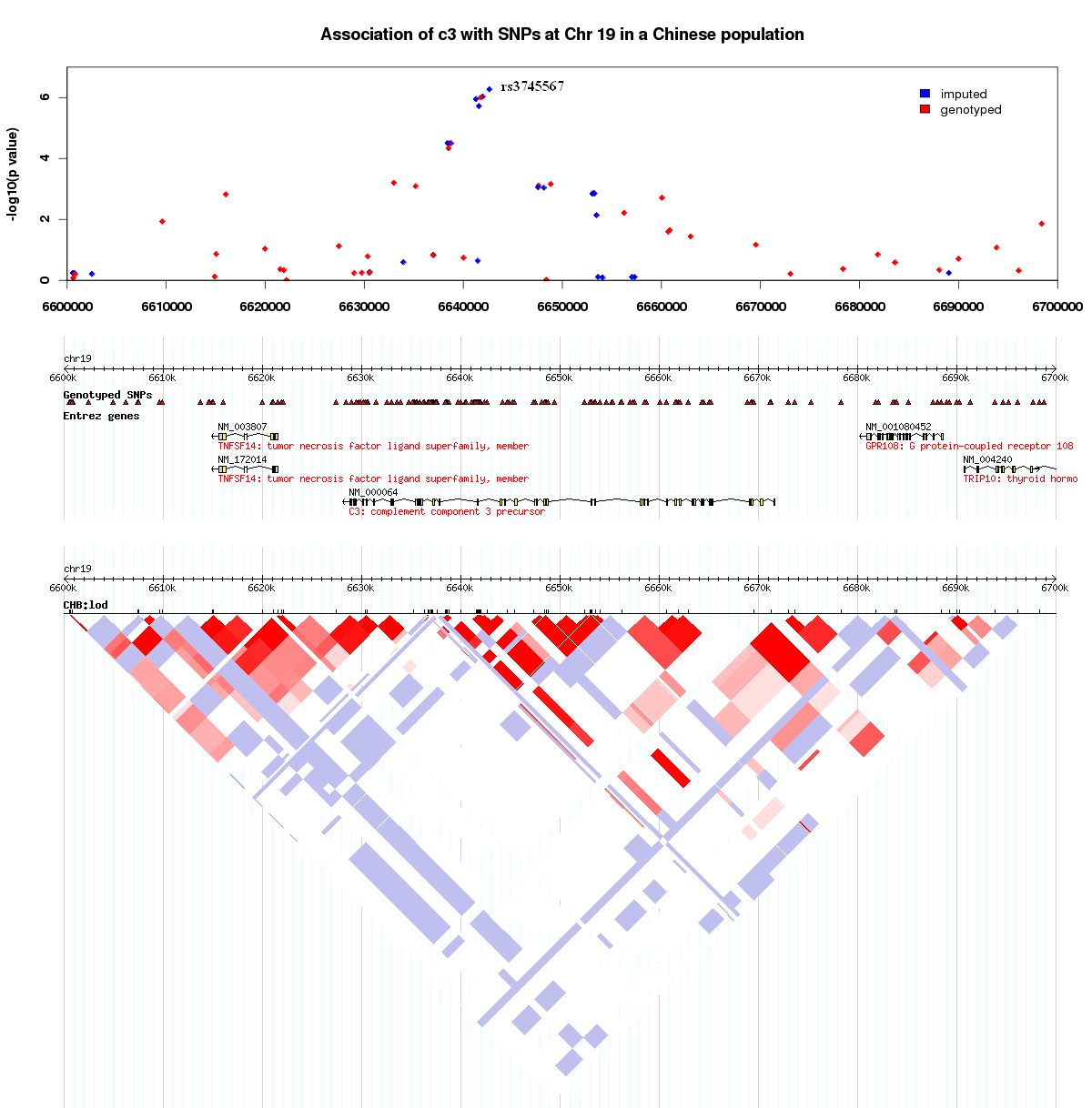

Supplement: Figure S4 — Association of serum C3 levels with SNPs at 1q32 and 19p13.3. The top panel presents association results in the first stage of GWAS, the middle panel describes genes in the region based on the UCSC database, and the bottom panel describes LD of SNPs in the region based on the HapMap CHB population (release 24). (DOC) [file pgen.1002916.s004.doc]

**Figure S5**

**(A)**


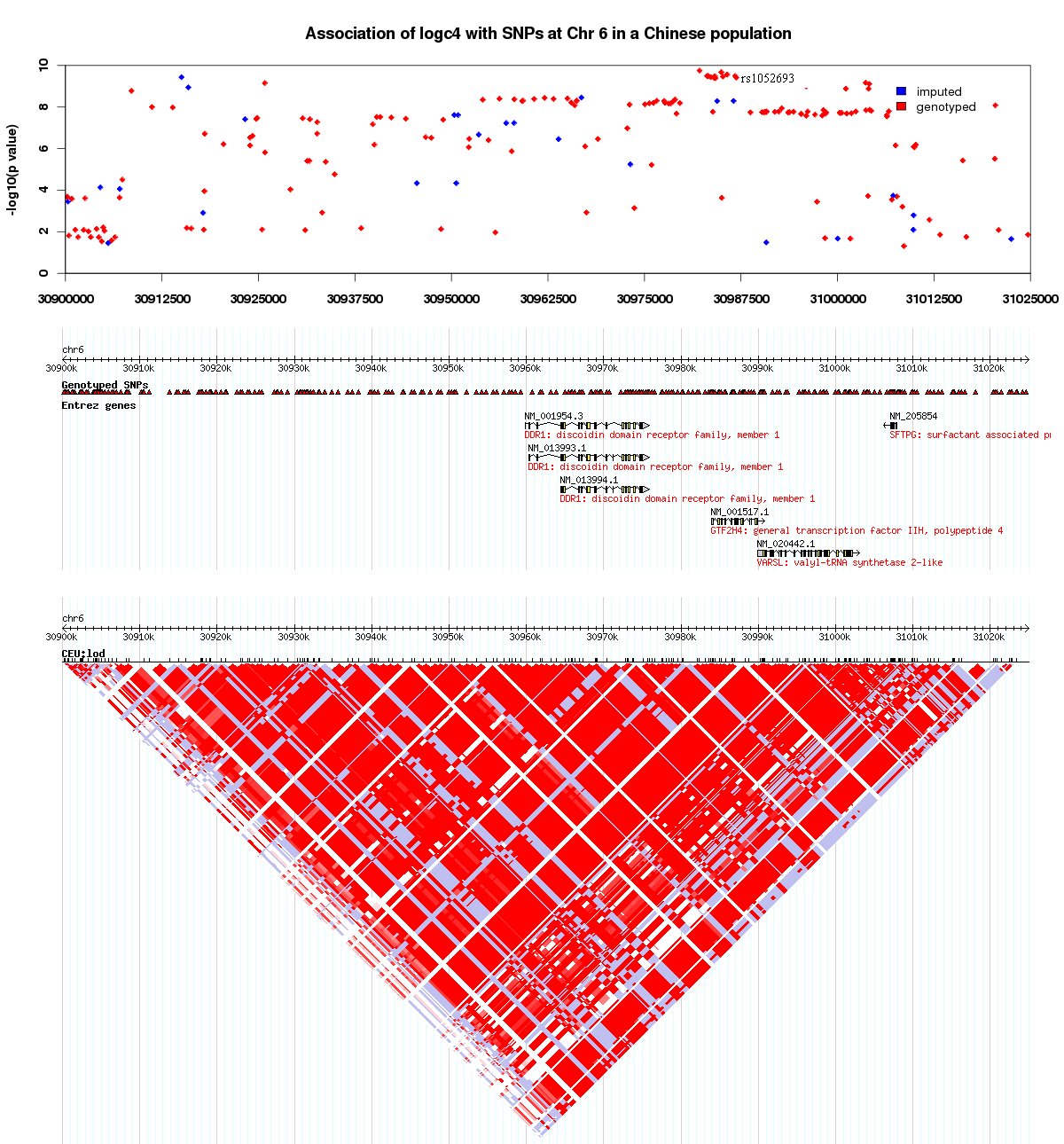


**(B)**

**
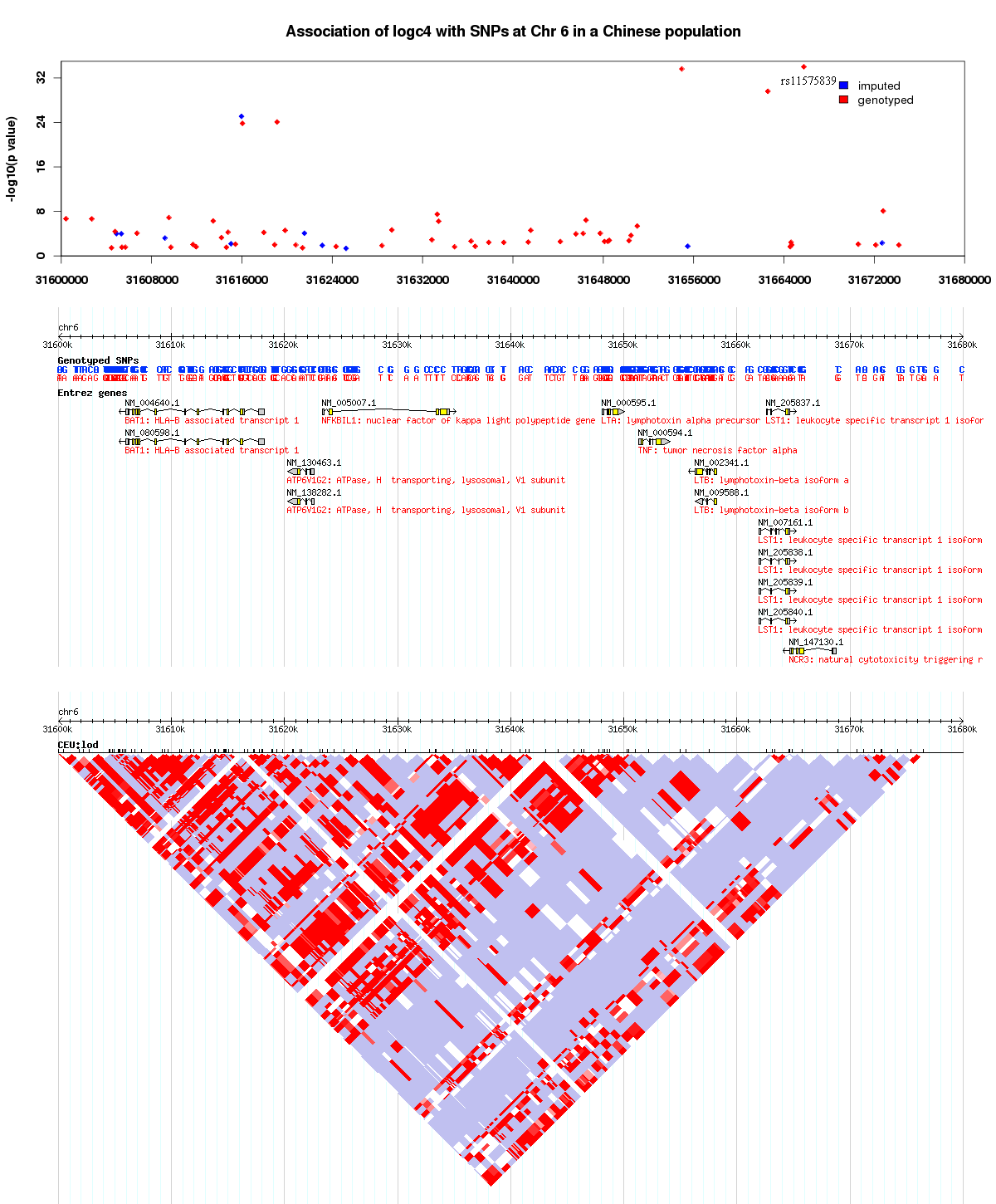
**

**(C)**

**
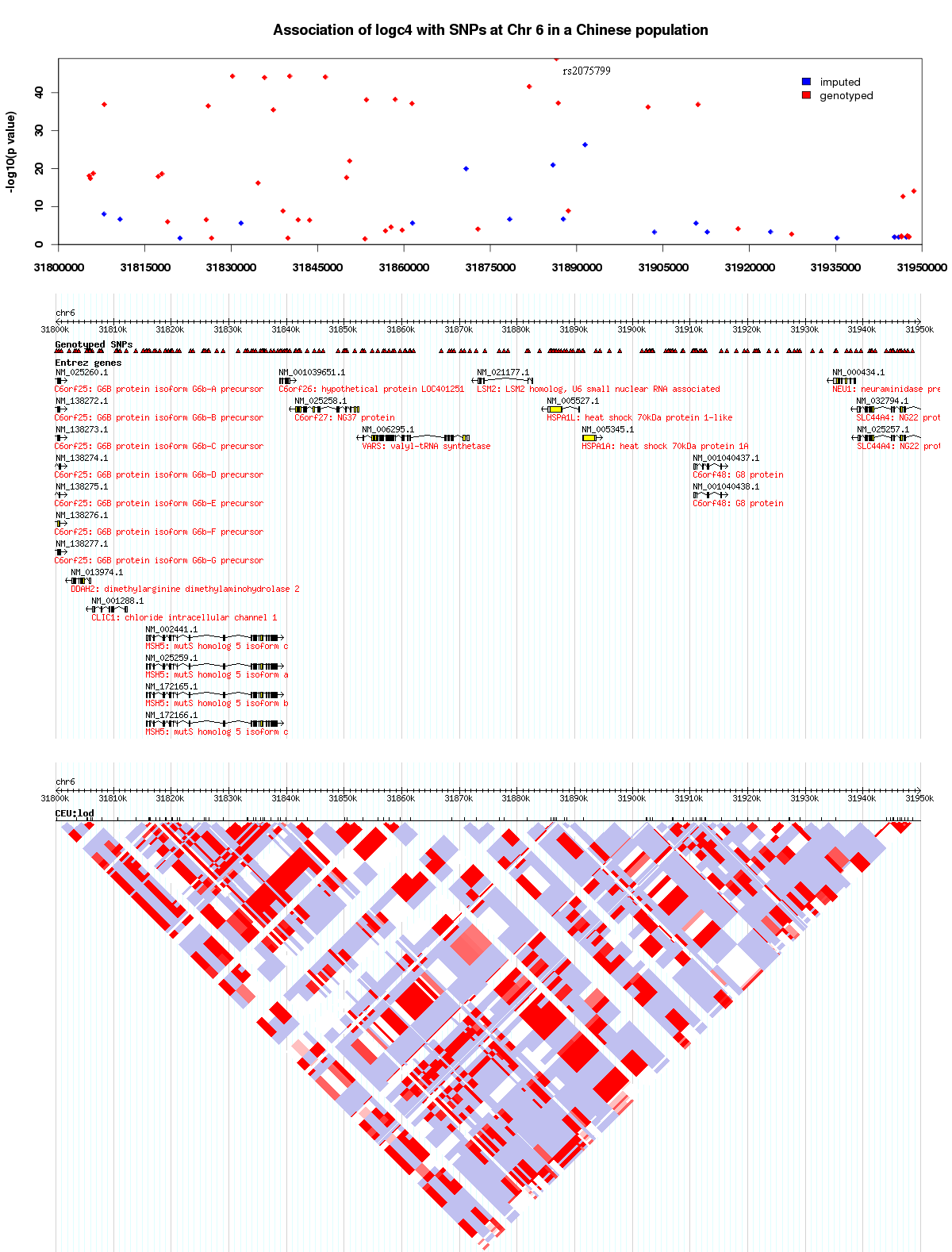
**

**(D)**

**
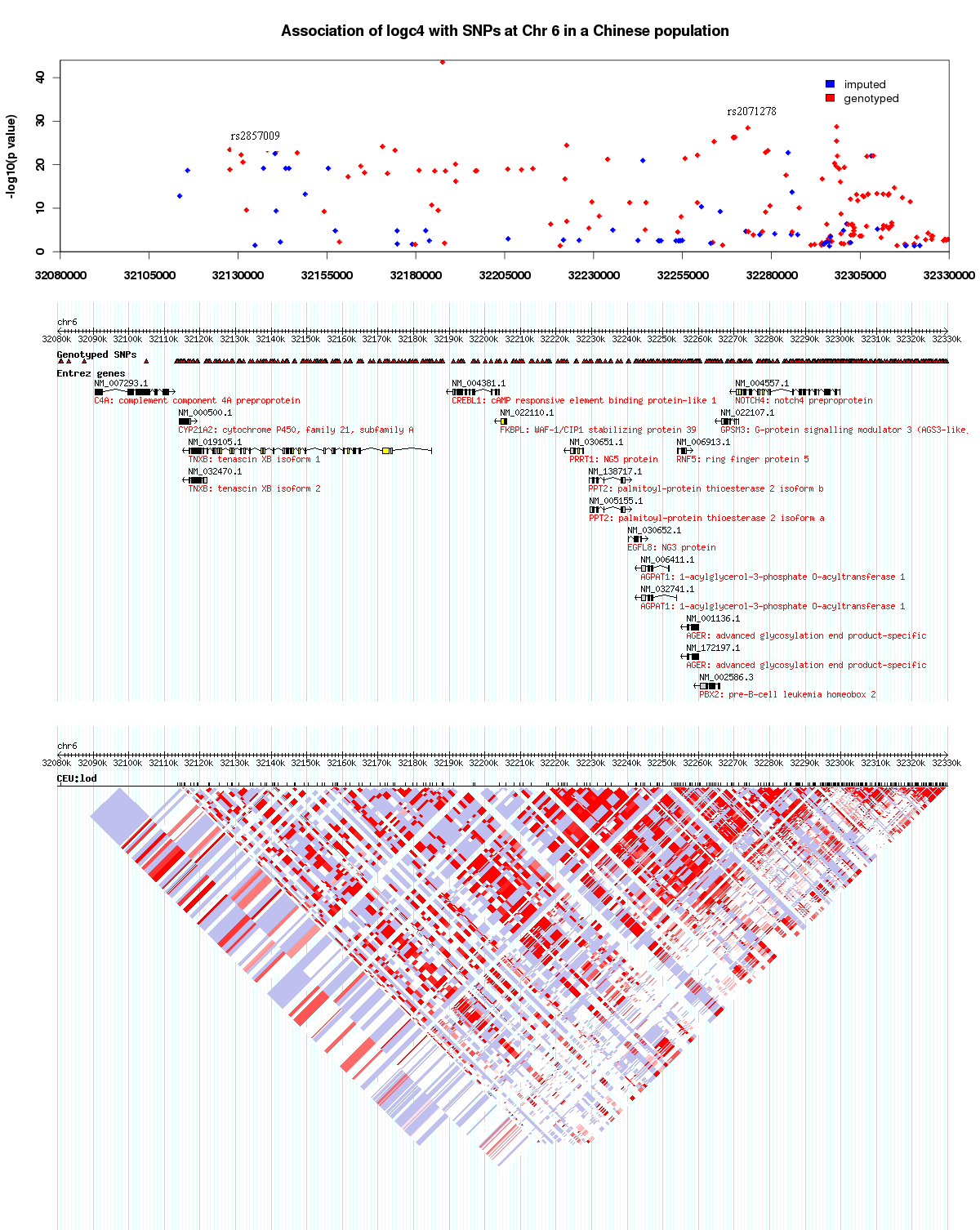
**

**(E)**


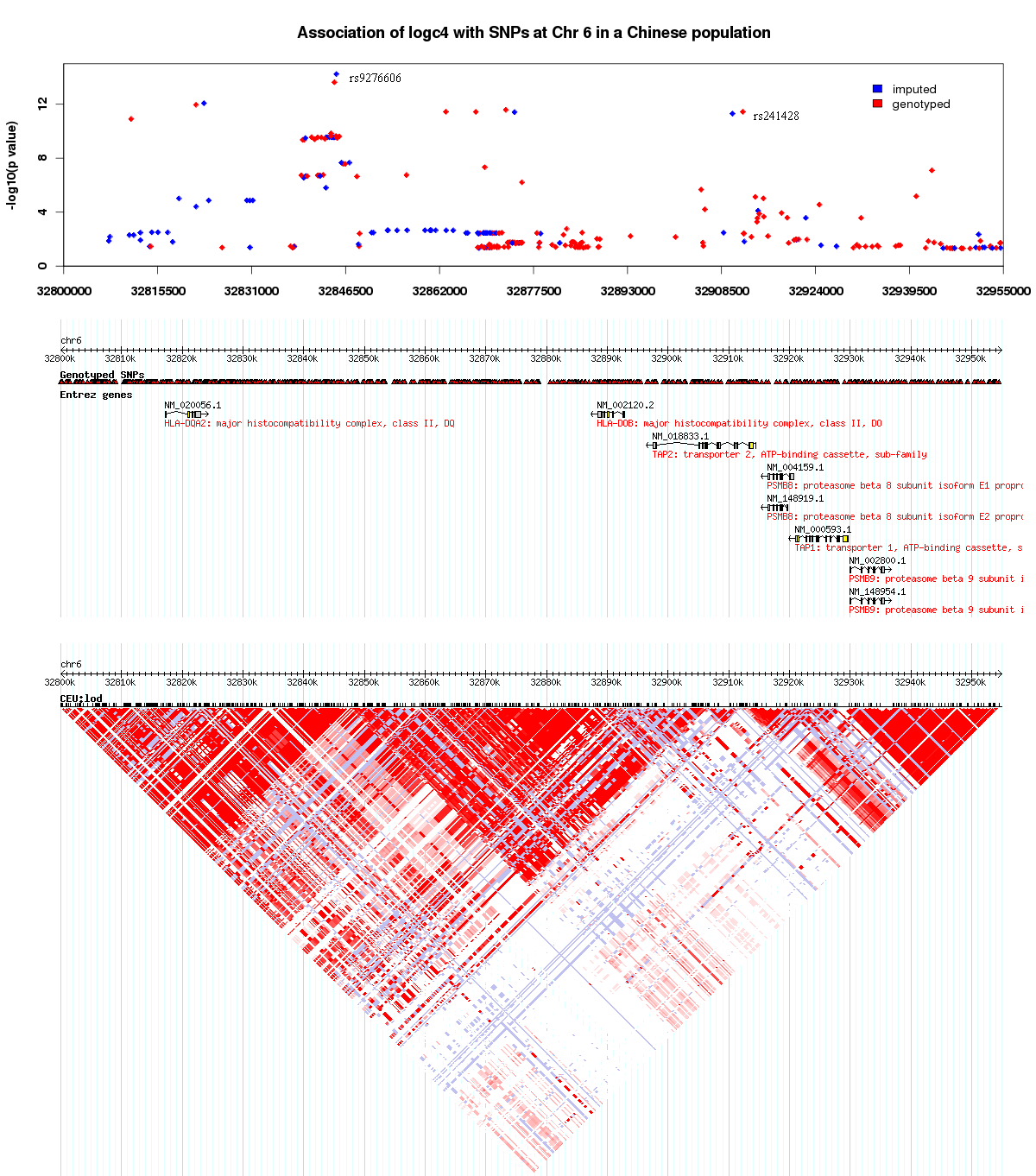

Supplement: Figure S5 — Association of serum C3 levels with SNPs at 6p21.3. The top panel presents association results in the first stage of GWAS, the middle panel describes genes in the region based on the UCSC database, and the bottom panel describes LD of SNPs in the region based on the HapMap CHB population (release 24). (DOC) [file pgen.1002916.s005.doc]
