# Supplementary material for: Genome-Wide Association Study for Serum Complement C3 and C4 Levels in Healthy Chinese Subjects
Source: PLoS Genet. 2012 Sep 13;8(9):e1002916. doi: 10.1371/journal.pgen.1002916 (PMC3441730; doi:10.1371/journal.pgen.1002916)
Supplement: Table S1 — SNPs associated with C3 and C4 levels from two-stage GWAS study in Chinese population, adjusting for chronic reactive protein (CRP). (DOC) [file pgen.1002916.s006.doc]

Table S1. SNPs associated with C3 and C4 levels from two-stage GWAS study in Chinese population, adjusting for chronic reactive protein (CRP)

| SNPs | Chr (Position) ^a^ | Genes | Gene  regions | Minor Allele/Major Allele | First Stage (N=1999) | | | | |  | | Second Stage (N=1496) | | | | |  | Combined (N=3495) | |
| --- | --- | --- | --- | --- | --- | --- | --- | --- | --- | --- | --- | --- | --- | --- | --- | --- | --- | --- | --- |
|  |  |  |  |  | MAF | Mean levels (g/L) | | | *P* value ^c^ |  | MAF | | Mean levels (g/L) | | | *P* value ^c^ |  | β(SE) ^c^ | *P* value ^c^ |
|  |  |  |  |  |  | aa ^b^ | Aa | AA |  |  |  |  | aa | Aa | AA |  |  |  |  |
| C3 |  |  |  |  |  |  |  |  |  |  |  | |  |  |  |  |  |  |  |
| rs3753394 | 1(194887540) | *CFH* | promoter | C/T | 0.43 | 1.16 | 1.13 | 1.08 | 1.15×10^-15^ |  | 0.44 | | 1.31 | 1.26 | 1.20 | 1.63×10^-3^ |  | -0.05(0.01) | 2.49×10^-11^ |
| rs3745567 | 19 (6641771) | *C3* | intron | T/C | 0.06 | 0.93 | 1.06 | 1.13 | 8.40×10^-10^ |  | 0.07 | | 1.07 | 1.16 | 1.26 | 2.90×10^-4^ |  | 0.09(0.01) | 6.33×10^-10^ |
| C4 ^d^ |  |  |  |  |  |  |  |  |  |  |  | |  |  |  |  |  |  |  |
| rs1052693 | 6 (30984131) | *GTF2H4* | 5'UTR | G/A | 0.36 | 0.28 | 0.31 | 0.34 | 8.52×10^-29^ |  | 0.34 | | 0.29 | 0.31 | 0.35 | 5.51×10^-23^ |  | 0.10(0.01) | 5.48×10^-48^ |
| rs11575839 | 6 (31665770) | *NCR3* | exon | A/G | 0.05 | 0.47 | 0.40 | 0.31 | 3.79×10^-41^ |  | 0.06 | | 0.41 | 0.41 | 0.32 | 5.98×10^-20^ |  | -0.23(0.01) | 1.83×10^-54^ |
| rs2075799 | 6 (31886508) | *HSP70* | exon | T/C | 0.20 | 0.40 | 0.36 | 0.30 | 2.47×10^-57^ |  | 0.20 | | 0.47 | 0.36 | 0.30 | 4.33×10^-50^ |  | -0.17(0.01) | 2.12×10^-99^ |
| rs2857009 | 6 (32127724) | *TNXB* | intron | G/C | 0.20 | 0.28 | 0.31 | 0.33 | 9.43×10^-15^ |  | 0.21 | | 0.28 | 0.31 | 0.34 | 8.88×10^-11^ |  | 0.08(0.01) | 5.11×10^-23^ |
| rs2071278 | 6 (32273422) | *NOTCH4* | intron | G/A | 0.27 | 0.38 | 0.34 | 0.30 | 8.54×10^-39^ |  | 0.28 | | 0.41 | 0.35 | 0.30 | 4.38×10^-39^ |  | -0.13(0.01) | 8.00×10^-72^ |
| rs3763317 | 6 (32484766) | *BTNL2* | 5'near | C/T | 0.37 | 0.28 | 0.31 | 0.35 | 3.77×10^-36^ |  | 0.38 | | 0.28 | 0.32 | 0.38 | 7.98×10^-34^ |  | 0.12(0.01) | 2.15×10^-68^ |
| rs9276606 | 6 (32844673) | *u/a* | intergenic | T/A | 0.15 | 0.39 | 0.34 | 0.31 | 3.29×10^-16^ |  | 0.15 | | 0.39 | 0.35 | 0.32 | 6.74×10^-11^ |  | -0.09(0.01) | 6.29×10^-24^ |
| rs241428 | 6 (32912048) | *TAP2* | intron | G/T | 0.08 | 0.22 | 0.26 | 0.33 | 1.77×10^-49^ |  | 0.09 | | 0.18 | 0.27 | 0.34 | 2.59×10^-38^ |  | 0.23(0.01) | 6.39×10^-86^ |

a. Genomic position is based on NCBI build 36.

b. aa indicates serum complement levels for homozygous carriers of minor alleles, Aa indicates for heterozygous carriers, and AA indicates for homozygous carriers of major alleles.

c. *P* values andβ-coefficient are based on multi-linear regression analysis for an addictive effect, and adjusted for age, smoking, logBMI, and serum loghsCRP. The combined *P*-values are calculated based on athe regression model, adjusting for the covariates and stage information.

d. The presented C4 levels were back-transformed.
