# Supplementary material for: Genome-Wide Association Study for Serum Complement C3 and C4 Levels in Healthy Chinese Subjects
Source: PLoS Genet. 2012 Sep 13;8(9):e1002916. doi: 10.1371/journal.pgen.1002916 (PMC3441730; doi:10.1371/journal.pgen.1002916)
Supplement: Table S2 — Correlation estimates between the significant SNPs with C4 copy number variation. (DOC) [file pgen.1002916.s007.doc]

Table S2. Correlation estimates between the significant SNPs with C4 copy number variation

|  | Pearson Correlation Coefficient^a^ | Single SNP P-value^b^ | Multi-variate P-value^c^ |
| --- | --- | --- | --- |
| rs1052693 | -0.29 | 0.043 | 0.936 |
| rs11575839 | 0.25 | 0.020 | 0.342 |
| rs2075799 | 0.36 | 0.271 | 0.337 |
| rs2857009 | -0.21 | 0.018 | 0.925 |
| rs2071278 | 0.34 | 0.026 | 0.640 |
| rs3763317 | -0.24 | 4.00E-04 | 3.00E-03 |
| rs9276606 | 0.24 | 0.026 | 0.505 |
| rs241428 | -0.29 | 2.49E-03 | 0.053 |

1. Pearson correlation coefficient was estimated for each SNP and copy number variation.
2. *P* values are based on single SNP analysis assuming an additive genetic model, adjusting for age, smoking, and logBMI.
3. *P* values are based on single SNP analysis assuming an additive genetic model, adjusting for copy number variation, age, smoking, and logBMI.
